# **“**Honestly, this problem has affected me a lot”: a qualitative exploration of the lived experiences of people with chronic respiratory disease in Sudan and Tanzania

**DOI:** 10.1186/s12889-023-15368-6

**Published:** 2023-03-13

**Authors:** Uzochukwu Egere, Elizabeth H Shayo, Martha Chinouya, Miriam Taegtmeyer, Jane Ardrey, Stellah Mpagama, Nyanda Elias Ntinginya, Rana Ahmed, El Hafiz Hussein, Asma El Sony, Tom Wingfield, Angela Obasi, Rachel Tolhurst, The IMPALA Consortium, Emmanuel Addo-Yobo, Brian Allwood, Hastings Banda, Imelda Bates, Amsalu Binegdie, Adegoke Falade, Jahangir Khan, Maia Lesosky, Bertrand Mbatchou, Hellen Meme, Kevin Mortimer, Beatrice Mutayoba, Louis Niessen, Jamie Rylance, William Worodria, Heather Zar, Eliya Zulu, Jeremiah Chakaya, Lindsay Zurba, S Bertel Squire

**Affiliations:** 1 Department of International Public Health, Liverpool School of Tropical Medicine, L3 5QA Liverpool, UK; 2 National Institute for Medical Research, Dar es Salaam, Tanzania; 3 Faculty of Education, Liverpool School of Tropical Medicine, Liverpool, UK; 4 Tropical Infectious Diseases Unit, Royal Liverpool University Hospitals NHS Foundation Trust, Prescot Street, Liverpool, UK; 5 Kibong’oto Infectious Diseases Hospital, Mae Street, Kilimanjaro, Tanzania; 6 Kilimanjaro Christian Medical University College, Kilimanjaro, Tanzania; 7 NIMR-Mbeya Medical Research Center, Mbeya, Tanzania; 8 The Epidemiological Laboratory, Khartoum, Sudan; 9 Department of Clinical Sciences, Liverpool School of Tropical Medicine, Liverpool, UK; 10 Department of Global Public Health, WHO Collaborating Centre on Tuberculosis and Social Medicine, Karolinska Institute, Stockholm, Sweden; 11 Honorary Consultant in HIV and Genitourinary Medicine, AXESS Clinic, The Royal Liverpool University Hospitals NHS Foundation Trust, Liverpool, UK

**Keywords:** Socioeconomic, Chronic respiratory disease, Stigma, Healthcare seeking, Psychosocial

## Abstract

**Background:**

Over 500 million people live with chronic respiratory diseases globally and approximately 4 million of these, mostly from the low- and middle-income countries including sub-Saharan Africa, die prematurely every year. Despite high CRD morbidity and mortality, only very few studies describe CRDs and little is known about the economic, social and psychological dimensions of living with CRDs in sub-Saharan Africa. We aimed to gain an in-depth understanding of the social, livelihood and psychological dimensions of living with CRD to inform management of CRDs in Sudan and Tanzania.

**Method:**

We conducted 12 in-depth interviews in 2019 with people with known or suspected CRD and 14 focus group discussions with community members in Gezira state, Sudan and Dodoma region, Tanzania, to share their understanding and experience with CRD. The data was analysed using thematic framework analysis.

**Results:**

People with CRD in both contexts reported experiences under two broad themes: impact on economic wellbeing and impact on social and psychological wellbeing. Capacity to do hard physical work was significantly diminished, resulting in direct and indirect economic impacts for them and their families. Direct costs were incurred while seeking healthcare, including expenditures on transportation to health facility and procurement of diagnostic tests and treatments, whilst loss of working hours and jobs resulted in substantial indirect costs. Enacted and internalised stigma leading to withdrawal and social exclusion was described by participants and resulted partly from association of chronic cough with tuberculosis and HIV/AIDS. In Sudan, asthma was described as having negative impact on marital prospects for young women and non-disclosure related to stigma was a particular issue for young people. Impaired community participation and restrictions on social activity led to psychological stress for both people with CRD and their families.

**Conclusion:**

Chronic respiratory diseases have substantial social and economic impacts among people with CRD and their families in Sudan and Tanzania. Stigma is particularly strong and appears to be driven partly by association of chronic cough with infectiousness. Context-appropriate measures to address economic impacts and chronic cough stigma are urgently needed as part of interventions for chronic respiratory diseases in these sub-Saharan African contexts.

**Supplementary Information:**

The online version contains supplementary material available at 10.1186/s12889-023-15368-6.

## Background

Chronic respiratory diseases (CRDs) are among the most common non-communicable diseases worldwide and include diseases such as asthma, chronic obstructive pulmonary diseases (COPD), bronchiectasis, occupational lung diseases and pulmonary hypertension [1]. Over five hundred million people globally live with CRDs [2].Approximately four million of these die prematurely annually, most of whom are from low- and middle-income countries (LMICs) [3]. Asthma and COPD share similarities in clinical symptoms and are by far the most common CRDs worldwide, affecting around 330 million and 200 million respectively globally [4]. In LMICs, the capacity for diagnosis and management of these conditions such as spirometry and inhaled corticosteroids, are generally limited in the primary care setting [1]. This further worsens the morbidity and mortality attributable to CRDs in these settings. In sub-Saharan Africa (SSA), CRDs are mainly caused by indoor air pollution due to domestic use of biomass fuel, which is used for cooking by approximately 90% of rural households [5]; tobacco smoke; and post tuberculosis lung disease [6]. Despite being increasingly recognised, CRDs are rarely prioritised by communities, health systems, or governments and robust data with which to inform interventions are lacking [7]. Reliable data on the prevalence of CRDs in Sub Saharan Africa are scarce. A systematic review [8] estimated the prevalence of COPD in Sub Saharan Africa to be 13.4%, similar to Western European and North American settings [9]. Asthma incidence has been estimated to be approximately 10% in Khartoum, Sudan [10], and recent data has shown a COPD prevalence of 16.5% in urban Sudanese adults who underwent spirometry [11]. Similarly, a study in Tanzania showed an asthma prevalence of 17.6% in adolescents from urban areas [12].

The psychosocial burdens associated with CRDs have long been recognised. For instance, poor asthma control has been shown to be associated with feelings of stigma [13]. Similarly, patients with chronic obstructive pulmonary disease reported feeling of ‘being exiled in the world of the healthy’ because of self-blame and society’s stigmatization of COPD as a self-inflicted disease [14]. This is similar to the well documented experience of patients with a communicable lung disease – pulmonary tuberculosis (TB). For instance, in India, female patients with TB were rejected by their families and up to 11% of children of these patients discontinued their studies with some taking up employment to support their family’s finances [15]. TB patients have also been reported to experience stigma and social exclusion in their communities [16].A systematic review showed that families of TB patients in LMICs experienced catastrophic costs, with approximately 50% of the costs incurred before treatment [17]. People living with CRDs incur direct costs of seeking care and indirect and opportunity costs such as costs associated with lost days of work, and a negative impact on quality of life [2, 18]. Most of the studies on the social and economic consequences of CRDs have however been conducted in high income countries and revealed, among other impacts, reduced workforce participation and employment rates, changes in employment or job duties as an adjustment to the disease, asthma-related lost work days, and impaired work effectiveness while on the job [19]. Very little is known about the social dimensions of CRD in LMICs, including stigma and social isolation, and there have been very few studies of the economic impacts on patients despite the heavy burden of CRD morbidity and mortality [20]. Studies exploring social and psychological dimensions of CRD are therefore urgently needed in LMICs, including SSA. An observational study of the socioeconomic impacts of CRDs in low-resource settings reported a median overall work impairment due to CRD of 30% among employed patients [21]. This was associated with substantial disease-related productivity and activity impairment among patients. However, this study focussed only on spirometry-diagnosed COPD and/or asthma patients. The lack of diagnostic and care pathways for CRD means there are many symptomatic people without a diagnosis or consistent care. More exploratory in-depth understanding of the circumstances, responses, attitudes and actions of patients and their communities is required to provide a broader context for addressing CRDs within the health systems in SSA.

We aimed to gain an in depth understanding of the economic, social, and psychological dimensions of living with CRDs for patients, their families, and communities, to support the development of context-specific interventions to integrate CRD care into routine health systems in Sudan and Tanzania. Rather than restrict our investigation to people with a confirmed CRD diagnosis we focused on people with chronic respiratory symptoms who had been investigated for TB and found not to have TB. Symptoms of wheeze, breathlessness and cough are easily understood by patients and communities and are presenting features when people with CRD seek care. This approach was used to facilitate research into community perceptions and individual lived experiences of CRD in communities where diagnoses are uncommon.

## Methods

We used qualitative methods to explore the perceptions, experiences and priorities of community members and people with chronic respiratory symptoms (including known or suspected chronic respiratory disease) to gain an in-depth, ‘emic’ understanding of their interpretation and experience of CRD. We used qualitative methods because they are best suited to answer questions about experience, meaning and perspectives from the standpoint of the participant [22]. We used an interpretivist approach [23] in order to allow participants to share their understanding and interpretation of CRDs and how these meanings influenced their experiences. We chose in depth interviews (IDIs) with people suspected of, or diagnosed with, CRD and focus group discussions (FGDs) with a representative group of community members. The constitution of the FGDs into same gender and age groups encouraged open discussions and allowed biases and beliefs to be freely discussed.

### Study sites and context

This study was nested in the multidisciplinary “International Multidisciplinary Programme to Address Lung Health and TB in Africa (IMPALA)” consortium, which focused on generating knowledge and implementable solutions for CRDs in Sub Saharan Africa. Sudan and Tanzania were selected for the IMPALA’s health systems research work package. Our baseline situation analysis in Dodoma region of Tanzania and Gezira state in Sudan characterised the health systems and explored the readiness of health facilities in these areas to deliver care for CRD [7]. The analysis found that most health facilities studies were inadequately prepared to manage CRD within their health systems. In Sudan, primary health services are provided at health centers in urban and rural settings, while district hospitals provide secondary as well as referral services within States. National hospitals and State teaching hospitals provide tertiary care. In Tanzania, primary health care services are provided at the ward/village level by health centers and dispensaries. Secondary health services are provided at the district level by the district hospitals while regional hospitals provide specialist referral services. Perspectives of policy makers and service providers have been published elsewhere [24, 25]. Both Sudan and Tanzania sites are mainly rural and semi-urban (according to local governments’ definitions), with inhabitants engaged mostly in informal livelihoods. Most study participants rely mainly on farming, informal income generating activities such as petty trading or working as masons, and relatively low paid formal work such as office cleaning. The sites were selected in collaboration with the local Ministries of Health (MOH) because they were perceived to be exemplars of good practice by the MOH in each country that could be built on to develop CRD services. Gezira State in Sudan was selected because of an existing pilot programme offering integrated lung health services focusing on asthma standard case management [26]. These services were developed by the IMPALA collaborating institution, Epidemiological Laboratory (EPILAB), a research non-governmental organization (NGO) with research infrastructure in Gezira. Dodoma Region in Tanzania was selected due to an embedded regional TB Control Program infrastructure and well-functioning community-based referral and follow-up system for people with chronic cough suspected to have TB even when their TB investigations are negative. The community referral system in Tanzania maintains contact with community members through designated community health workers who identify people with chronic cough who were found to be negative for TB but continue to seek care in the health facilities for on-going respiratory symptoms.

### Participant selection and recruitment

For IDIs, purposive sampling was used to select participants with confirmed or suspected CRD [27]. In Sudan, patients were purposively selected from the asthma standard case management programme at EPILAB sites to include men and women with varied asthma severity. Diagnosis of asthma was made by clinicians using diagnostic algorithms and peak flow meter measurements at the EPILAB sites. In Tanzania, the research team selected patients in collaboration with health workers in the catchment area health facility. In Tanzania, clients with presumed CRD who had been investigated for TB, were found to be negative but remained ill and continued to visit the health facility for treatment without a definitive CRD diagnosis, were purposively selected from catchment areas of study health facilities. In both countries, patients were approached face-to-face- and selected from catchment communities closer and farther away from the health facility, and with a balanced representation by gender and severity of symptoms. All participants approached agreed to participate.

For FGDs in both Sudan and Tanzania, community representatives were selected to reflect the range of literacy levels and socioeconomic backgrounds within the community in both countries. In Sudan, an additional group of adolescent girls was constituted to create a socially safe and trusting environment for open discussion, based on the research team observations that young women and adolescent girls were particularly likely to experience negative social impact of asthma in the community. Presence or absence of CRD was not a criterion for selection of community representatives in the FGD.

### Data collection

We used common data collection methods across the two countries. The topic guides used in the in-depth interviews and focus group discussions in this study were specifically developed for this study. All data were collected in 2019, from January to February in Tanzania and February to August in Sudan, prior to the COVID-19 pandemic. Topic guides were used to guide semi-structured interviews and focus group discussions (additional files 1 and 3) and explored patients’ and community understanding of CRD, impact of CRD on productivity, work life, finances, coping mechanisms, experiences with CRD and priorities for care. Additionally, in Sudan, patients’ perceptions and experiences with the asthma management services were explored in semi-structured interviews using a topic guide (additional file 2).

Interviews and FGDs were conducted by researchers employed by EPILAB (Sudan) and National Institute for Medical Research (NIMR) Tanzania and experienced in qualitative research; in Tanzania by ES, (female, PhD), and in Sudan by EH (male, MSc) with support from trained research assistants (both male and female). Training of research assistants already experienced in qualitative research was conducted in each study site and covered overall aims of the study, data quality, ethical issues in health facility research, informed consent, privacy, and confidentiality. Training included hands-on practical sessions on qualitative interviewing, adapting topic guides and a data collection pilot in the community. The pilot data was analysed and used to inform data collection in the main study.

Interviewees were not known to interviewers before the studies commenced. During informed consent processes, interviewers were introduced as professional researchers working for their respective organizations and the purpose of the study was fully explained. Interviews were audio recorded and conducted in the local language (Swahili in Tanzania and Arabic in Sudan) in a quiet, private, and secure room within the health facility premises. Each interview lasted approximately 60 min. FGDs were conducted in mutually agreed venues in the communities and each one lasted about 90 min. Light refreshments were provided to all participants at the end of the interviews and FGDs. No financial compensations were offered. A note taker took notes during the interviews and a debrief session was observed after interviews to reconcile any inconsistencies. No repeat interviews were conducted. Transcripts were discussed with participants to ensure understanding where necessary. All interviews were transcribed by the field team, quality checked and translated into English by professional translators prior to data analysis.

### Data analysis

We used the Framework Method [28] for the management and analysis of data as it facilitates transparent analysis and allows teams of researchers to collaborate on analysis [29]. This method identifies commonalities and differences in qualitative data, and then focuses on relationships between different parts of the data in order to draw descriptive and/or explanatory conclusions around identified themes. To improve trustworthiness, five members of the research teams (UE, ES, EH, MC, RT) independently read and re-read manuscripts, inductively identifying emerging codes and developed an analytical coding framework for coding the rest of the data after reviewing a few transcripts together and resolving discrepancies. This process was iteratively repeated till no new codes emerged. Separate coding frameworks were developed for patient interviews and focus group discussions. Codes were organised into broader categories, which were used to chart data, bring data from each country into a single spreadsheet matrix. Similarities in data from interviews and group discussions enabled the use of common categories at this stage. Within this framework, experiences of female and male interviewees were compared with a consideration of other influencing factors such as diagnostic status. Experiences related to aspects of gender relations were identified within the accounts using concepts and categories from a gender analysis framework, which were included within the overall analytical framework [30]. The chart was used to identify overall themes emerging from the data. We collectively judged that thematic saturation was reached with some limitations related to the impact of the political unrest in Sudan during data collection (see note on Table 1). It was not possible to conduct participant checking discussions following analysis due to onset of political unrest in Sudan and the covid pandemic. This paper presents emergent themes related to the social, livelihood and psychological dimensions of living with CRDs.

**Table 1:** FGD location and characteristics of FGD participants

FGD location	Age group	Gender	Number of participants
Sudan			
Al-Musalmia	> 30	Male	7
	> 30	Female	7
Masoudia*	15—17	Female	10
Rihanna*	15—29	Male	9
Sarasirr	> 30	Male	9
	> 30	Male	8
Tanzania			
Mvumi	18 – 30	Male	9
	18—30	Female	10
Chamwino	> 30	Male	9
	> 30	Female	11
Mpwayungu	18—30	Male	8
	18—30	Female	10
Haneti**	> 30	Male	9
Dabalo	> 30	Female	12
Total			128

^*^Only one FGD conducted due to nationwide political unrest

^**^ Haneti and Dabalo have similar demographic characteristics

## Results

### Demographics of study participants

We conducted 12 in-depth interviews: 7 in Tanzania (3 males, 4 females) and 5 in Sudan (2 males, 3 females). Of the participants from Sudan, two had secondary school education, one was a secondary school student whilst two (both women) had no education at all. Of the two without education, one was a housewife and the other was a cleaner. Of the participants from Tanzania, two (both men) had secondary school education whilst all others had only primary education. Of the women, one was a food vendor, one a petty trader and two were subsistence farmers. One of the men was a subsistence farmer, one was a brick layer, whilst one was a painter. The location and composition of the FGDs, and the number, age, and gender of the participants, are shown in Table 1 below. There were 128 FGD participants, 50 in Sudan and 78 in Tanzania, with each FGD group consisting of 7–12 participants. Youth participants were defined as those aged less than 18 years.

Participants described how CRDs impacted their activities, relationships, interactions within the community, and overall wellbeing. The results are presented in two broad themes: impact on economic wellbeing and impact on social and psychological wellbeing. Economic impacts included limitations on livelihoods and impacts of healthcare seeking costs and CRD mortality on patients and their families. Social and psychological impacts resulted from social exclusion, discrimination, stigma and impacts on mental health, and sexuality.

### Impact on economic wellbeing

Respondents with CRD in the various communities reported limitations on their livelihoods resulting from diminished capacity to do hard physical work. This was similar in both countries for women and men as both relied largely on physical work. For most people this was due to being unable to be as ‘productive’ in farming or informal work, but for some this involved reduced working hours in formal employment. Many people with CRD had to stop work to attend clinics, be hospitalised, recuperate after treatment and/or attend follow-up appointments. This resulted in both direct and indirect economic impacts for people with CRD and their families due to lost working hours and inability to continue with physical work. While some people with CRD could change their work to involve less vigorous and less physically demanding tasks, others stopped working entirely.

“I have been affected terribly, especially in my daily responsibilities. Our job, especially we Tanzanians, most of our activities are heavy duties, most of the activities are performed in the sun, most of them are in dusty working conditions on the land. I find it difficult, I can’t work effectively” (IDI, Male, Patient, Tanzania).

Subsistence farming was common in our study sites with any surplus produce being sold for cash. Therefore, reduced ability or inability to work impacts on both the family’s food supply and their cash income. Loss of income meant inability to pay school fees for dependent children and inability to take care of dependents, including aged or infirm older relatives. A female cleaner working in a local primary school reported stopping work when she developed CRD because of fatigue and breathlessness:

“In the past, I used to do any work and move around. Since I developed this lung problem, I stopped” (IDI, Female, Patient, Sudan).

A male construction worker lost working days to illness and reported a negative impact on employer perceptions of his ability: even when he made himself available to do some work:

“Honestly, this problem has affected me a lot because I can’t engage in activities to earn my income since I fear for the problem I am having. When you go to the sites looking for a job, you may miss for some days because of your health problem, someone looks at you and says; ‘This one can’t do this job’. Therefore, I am being affected in many ways such as taking care of myself and my family financially.” (IDI, male CRD patient, Tanzania).

Many people with CRD also reported several costs incurred while seeking healthcare, including expenditures on transportation to the health facility and procurement of diagnostic tests and treatments.

A teacher with asthma in Sudan reported huge transportation costs resulting from long distance travel to access specialist treatment:

“And even if I want to go to Khartoum or Madani to see a specialist, I'd need to pay 1000 SDG [2.4 USD] for transportation only, and that's almost my salary, let alone the doctor's fee and medications and tests” (IDI-Male, Patient, Sudan).

In both countries, transportation costs were especially high when the health facility was located far away from the patient’s community and this challenge was so significant that some community members suggested they would be willing to pay specialist consultation fees if the specialist could come to a health facility nearer to them, obviating the need for transportation. In some cases, people with CRD were reportedly accompanied on their multiple health facility visits and admissions by bread-winning family members who incur transportation costs and loss of working hours during these periods, which in turn impacts family livelihoods.

“The family will also be affected; they (relatives) will accompany the patient when s/he falls ill. All livelihood activities will be put on hold whether these activities are in the private or public work…The whole family will all be at hospital. Financially and physically, they will be exhausted." (FGD, Female, Sudan).

Participants described multiple visits to seek care in different health facilities. This journey would usually start with the local pharmacy and may end up in high level health facilities often located far away from the person with CRD. This involved not only transportation costs but also costs of medications and tests which are often beyond the ability of the person with CRD to afford. A participant described treatment costing an entire month’s salary:

“I'm dependent on my salary, but medications are very expensive and so is the doctor's appointment, in addition to the original expenses of life in general. Some way or another it'll affect you because the cheapest medicine is worth a month's salary.” (IDI, Female, Patient, Sudan).

The death of a relative with CRD was reported to impact the economy of the extended family in Tanzania. A participant reported that, in the event of death of a patient, relatives would be expected to take over their responsibilities such as care of the family, parenting, payment of school fees, provision of basic needs and management of assets, all of which exert major economic pressures on the patient’s relatives.

“I had a relative who had such a problem, we came to realize when it was too late, and she had a family. When she felt sick, she used to go to the hospital to perform some tests and get medication. In the end, they realised that her lungs were badly damaged. Unfortunately, she died, and we, as relatives, have started helping and supporting the kids left behind for their education. So, it affects the family” (FGD, female adult, Tanzania).

### Impact on social and psychological well-being

Many participants described a sense of exclusion from community social life related to their inability or reduced ability to participate in routine personal and community activities such as going to work, attending social gatherings, and doing household chores and cooking. A female patient in Sudan reflected on limitations on her social activities after being discharged from hospital following an exacerbation of asthma:

“You see now after the two months I spent at the hospital, whenever I go to an occasion I’ll only participate in the conversation, I can’t help and serve as I used to. I used to help with cooking the food and washing the dishes, but now even the conversation might start a coughing seizure for me” (IDI-Female, Patient, Sudan).

Similarly, in Tanzania, a male patient expressed frustration at his inability to participate in the digging of grave, an important activity for men when there is a death in the community.

“I may participate in community activities by just being there and see what is going on, but I can’t fully participate because, for instance, there might be a funeral, I attend the funeral, but I can’t participate in digging a grave.” (IDI, Male, Patient, -Tanzania).

In all study communities, male and female participants in both countries reported experiences consistent with stigma as a major social impact of CRD and these resulted in part from association of chronic cough with infectiousness. Stigma was both enacted (actions of discrimination by others) and internalised (withdrawal by affected individual because of their own negative feelings or anticipation of others’ negative responses) and resulted in isolation from peers and the wider community.

In Tanzania, some female participants with CRD described how they excluded themselves from community gatherings such as the market or church, as chronic cough is mostly associated with TB and HIV in the community and raised concerns about transmitting infections among members of the community.

“I used to go to church, but when I went there, I was coughing a lot, and everybody used to look around to see who was coughing frequently? I decided not to go [anymore]. Since [then], I have never gone to church” (IDI, Female, Tanzania).

Participants commonly spoke of chronic cough as infectious and described people with chronic cough being stigmatised because they were presumed to have TB or HIV. A male FGD participant in Tanzania highlighted this:

“Stigmatization exists here to a great extent because coughing is also a symptom of HIV/AIDS.” (FGD-Male-Mvumi-Tanzania).

Stigmatization here referred to people moving away from anyone coughing openly and frequently for a prolonged period.

Male and female participants reported avoiding people with CRD for fear of getting infected. For example, a male FGD participant referred to a scenario where people with CRD would be avoided by potential sexual partners due to fear of contracting HIV:

“If someone is coughing frequently, people would suspect him to have acquired HIV and they will fear him thinking he is HIV-positive, he will no longer participate in other issues. Even some girls would be fearing him and would not want to have sex with him” (FGD, male youth, Tanzania).

Similarly, in Sudan, asthma was believed to be infectious and people with CRD may be labelled as ‘TB’ sufferers because of chronic cough:

“For them, asthma is something infectious that needs to be treated or a disease that might be possible to cure. For instance, they tend to say that this person (is) living with tuberculosis or something else.” (IDI-Female-Sudan).

‘Them’ here refers to members of the community.

Some people with CRD including men and women mentioned that they did not disclose their illness to family members and the community. In Sudan, some people with CRD reported pretending to be well when in the company of other people, hiding their illness from family and friends because they feared being a burden to their neighbours or family. A young male with asthma highlighted how important it was to him for the family to be protected from feeling his ‘pain’:

“Yes it does…it hurts me a lot, but I don’t want my family to feel my pain.” (IDI-Male, Sudan).

Of particular importance to female FGD participants in Sudan was the potential for loss of marriage opportunities due to being discriminated against by potential suitors when a young woman developed CRD or tuberculosis. This is because of fears that CRDs such as asthma could be infectious like TB, and also that they could be passed from generation to generation. Marriage is a very important part of the identity of a woman in Sudanese society and a symbol of her status both of her family and her own position within it. A female FGD participant who spoke of the impact of CRD on young unmarried women, described the difficulties a young woman or girl with CRD would have in attracting a suitor:

“It will lead to single marital status. It will lower the market value.” (FDG, Female adult, Sudan).

The impact of CRD-related stigma on marriage was reported not only to be limited to the prospective wife but also to her family with the potential to jeopardise future marital prospects for other women in the family:

“It is a social problem, solely a social problem. [Even] if the girl is so beautiful like the moon, they will tell you not to marry from her family as they have so and so.” (FGD, Female, Sudan).

Similarly, a male participant emphasised that prospective husbands may be discouraged from marrying young women with asthma for fear that asthma may be passed on to the children:

“In another area, people start to avoid and say that person is contagious, this is a social part. They say X shouldn’t marry Y because she has asthma and may affect children after that, pulling them to those diseases” (FGD, Male, Sudan).

Participants also reported their inability to play the usual expected roles in the society had impacts on their sense of belonging and contributions to the society. This represents an important intersection between the economic, social and psychological impacts of CRD on the patient and their families. In both contexts, the social impacts of CRD such as impaired community participation and restrictions on social activity, led to psychological stress to both people with CRD and their families. Male and female participants with CRD used terminologies such as ‘moody’, ‘angry’, and ‘crying’ to describe the impact of CRD on their psyche. A patient in Sudan described himself as always in a bad mood and angry:

“I am always in a bad mood, and I feel exhausted to the point that I am not able to leave my bed. Very angry.” (IDI, Male, Sudan).

For others, crying was a frequent means of communicating feelings at home when overwhelmed with emotions because of CRD.

“Sometimes we communicate only in tears. I feel like I can't talk and just want to cry “(IDI, Female, Sudan).

The sense of uncertainty around the illness was reported to be shared by the entire family:

“if there is someone sick in the house, the whole of the family will be in stressful condition particularly if the patient could not breathe… The whole family will be anxious; not only the patient but all the family members will be in stressful condition.” (FGD, Female, Sudan).

The impact of CRD symptoms on sexual activity was a contributor towards negative impacts on wellbeing. Several male community members in Sudan emphasised that CRD symptoms could impact the desire and capacity for sexual activity, and this was gendered, as it was an important indicator of wellbeing and pride among men in the community.

“To get to the bottom of this issue, if I have the bad disease, I will be psychologically depressed. I have (something) in my head that I am going to die. I will not have the desire to sleep with my wife, reproduce or have children.” (FGD-Male-participant-Sudan).

The use of the term ‘bad disease’ here highlights the participant’s perception that chronic cough was ‘bad’ in line with the general perception that chronic cough was due to tuberculosis or HIV which could lead to death.

## Discussion

We found multiple economic, social and psychological dimensions of living with chronic respiratory disease on people diagnosed or suspected of having the disease, on their families and communities. Both CRD symptoms and treatment seeking resulted in an economic toll on people with CRD and their families in a range of ways, including impairment of physical fitness and ability to work, loss of working hours and jobs, and direct and indirect costs of health seeking such as costs of transportation to the health facility and treatment and opportunity costs of missed work. Our findings further show that stigma associated with chronic cough resulted in social exclusion. The social impacts including inability to carry out activities of daily living, impaired community participation. Non-disclosure to families and restrictions on sexual activity in the context of stigma and social exclusion, were found to be a source of significant psychological stress on people with CRD and their families.

The strong association of chronic cough with TB in these settings is not surprising. Chronic cough was widely presumed to be TB in a similar study among Ugandan communities [31]. Whilst Tanzania is one of the 30 high TB burden countries, Sudan has a moderate but significant burden of TB [32].The common knowledge of TB in the communities may be explained by the historicity of endemic TB disease and by the substantial investments in TB control compared to CRD and other diseases in these contexts. A downside of this association, as seen in this study, is the stigmatization and ostracism of people with CRD in the community. This may lead to failure to attend health facilities for fear of being confirmed as having TB disease, which further delays diagnosis and can lead to increased morbidity and mortality [16, 33]. Misinformation about chronic cough is another driver of stigma seen in this study. A study investigating knowledge and perceptions of asthma among secondary school students in Tanzania revealed that most information about asthma had been passed on to students by their parents and non- asthmatic students presumed that asthma was infectious and therefore avoided interactions with their asthmatic colleagues for fear of contracting the disease [12]. Systematic community sensitization activities with trained and well-informed health personnel could help improve community perception about chronic cough and contribute to tackling stigma in CRDs. Clear messages about the causes of asthma and the availability and effects of treatment on controlling symptoms would help to destigmatise asthma and other CRDs. A study in Kenya, a comparable context, found that the effect of seeing people being managed well with affordable medicines and transform their lives can be more powerful than being told about treatment effectiveness [34]. Words matter and consistent use of terms like ‘people who have asthma’ rather than asthma ‘victims’ or CRD ‘sufferers’ can start to shift mindsets. There are many transferable lessons to be learned from community de-stigmatisation efforts with HIV in the antiretroviral therapy era [34] and, more recently, efforts being applied to COVID-19 related stigma [35].

The economic impact of CRD in these communities highlights the vulnerability of people with CRD in mostly rural settings, who are self-employed or working in informal sectors of the economy such as subsistence farming, without recourse to any social security, and often with daily income reliant on physical work. Chronic diseases, including CRDs, are known to incur significant economic costs for both patients and the health system [36, 37]. In Malawi, another comparable context, the mean care seeking cost for chronic cough per patient was found to be 2.3 times the average cost per capita on health for the country and consisted mainly of transport and drug costs [38]. Similarly, TB-related catastrophic costs have been well recognised and documented. A systematic review of financial impact of TB in LMICs revealed that on average the total cost was equivalent to 58% of reported annual individual and 39% of reported annual household income. To tackle catastrophic costs in TB context, poverty reduction strategies are increasingly being woven into TB control programmes including social protection initiatives such as cash transfers, food baskets and social insurance [39]. Health systems could draw from these experiences and interventions in TB to develop an integrated approach to addressing the economic impacts of CRDs in the sub-Saharan African setting.

Transportation costs were strongly highlighted by our study participants as a major source of economic loss, impacting people with CRD and relatives who accompany them on hospital appointments. Our informants themselves stated that the transportation costs were often the equivalent of their monthly salary. Transportation has been identified as an important social determinant of health [40] and a well-documented barrier to engaging in the care of chronic diseases especially among poor, vulnerable populations [41]. Studies done in high income settings have shown that interventions such as provision of bus passes, taxi vouchers and reimbursements from insurance covers could improve healthcare utilization [42] but these interventions may not be feasible in our study setting because of lack of transport infrastructure. Policy makers in our study context could consider decentralisation of services and investment in specialist mobile clinics to rural areas and geographically distant communities as measures to reduce the impact of transportation costs and improve health outcomes of people with CRD. Mobile clinics have been successfully deployed in primary health care setting in Malawi [43] and Nigeria [44] targeting care of several common conditions within the rural society including malaria and respiratory illnesses. In our study setting, the extended family system and the social interconnectedness meant that any economic costs incurred by the person with CRD could lead to a ripple effect within their social circle, potentially setting up or exacerbating vicious cycles of poverty and ill-health. Life course studies, including in sub-Saharan Africa have established that risk factors in childhood and adolescence, including poverty, contribute to subsequent development of CRD in adulthood [45]. The economic impacts observed in this study risk perpetuating poverty and worsening outcomes such as morbidity and mortality. Measures to mitigate these impacts would be crucial in breaking the cycle of poverty and ill-health, improving outcomes in subsequent generations. Developing a clear diagnosis and care pathway for CRD in our study setting would be an important part of this mitigation.

Difficulties to engage in usual activities of daily living, disruptions in sexual activities and difficulties with community participation reported in this study have been shown to compound the negative impacts on wellbeing of chronic diseases including asthma [46], COPD [47] and HIV/AIDS [48]. Physical challenges of managing the disease can sometimes affect mood and emotional health leading to anxiety and depression in both people with CRD and their carers [49]. Health system responses to CRD should include supporting carers and families by telling them what to expect, encouraging them to present to care at the earliest recognition of symptoms and patient-centred communication to support with self-management of symptoms where appropriate. Research in other contexts shows that recognising and managing anxiety and depression can increase ability to stick with prescribed COPD treatment, improve physical health and reduce medical costs [50].

The finding in this study that young Sudanese women with chronic cough and their families were discriminated against by potential suitors further highlights the gendered nature of stigma associated with chronic cough. The perception that asthma, a major cause of chronic cough well known in this society, was hereditary, has also been described in a previous study of asthma in urban Sudan [51], where 67% of 490 asthmatic patients believed that asthma could be transmitted like an infectious disease within the family. The study also highlighted denial of asthma diagnosis and non-use of inhalers by young female asthmatic patients as a coping mechanism to avoid stigma. This highlights the disproportionate social burden of CRD borne by young women. A related finding in our study is the choice made by people with CRD to hide their symptoms from family and community members in a bid to keep the emotional burden of illness away from them. Young people with chronic illness in high income countries have been shown to be particularly circumspect about disclosure of their illness, frequently choosing non-disclosure because of perceived fear of rejection, pity, and perceptions of being seen as vulnerable or different [52]. In the context of stigma, non-disclosure could also be a coping mechanism to avoid stigmatization while shielding the family from its effects, as observed in our study.

### Limitations of the study

As this was a qualitative study, quantitative costs were not collected, and quality of life not measured. Quantification of economic impact through a cost of illness study would lay the foundation for a future evaluation of the cost-effectiveness of interventions to address CRD within the health systems in these settings. However, the direct testimony of the people with CRD and members of their communities about their lived experience, provides important information about the range of economic, social and psychological dimensions of living with CRD and insight into potential interventions to address them. Failure to complete the number of planned FGDs in Sudan might have limited thematic saturation but the impact of this on the overall results is likely to be small as the targeted age group and gender at the two sites were covered in the conducted FGDs. Comparing experiences across disease conditions would have been helpful but limited capacity to make accurate diagnosis in our study settings did not allow this. However, given the similarity in the clinical manifestation of CRDs, lessons learnt from the patients remain valid and can inform management across the different CRDs. Whilst the findings are not statistically generalisable to populations in Sudan and Tanzania because of purposive selection of relatively small number of communities, or sub-Saharan Africa more widely, the common issues arising within these two different contexts are likely to be generalisable to similar settings, whilst the context-specific issues point to the importance of locally developed interventions.

## Conclusion

Living with chronic respiratory diseases has a range of social, livelihood and psychological dimensions for people with CRD and their families in Sudan and Tanzania. While the impacts on livelihood and household economies resulted mainly from impairment of physical ability to carry out income-generating activities and from expensive healthcare costs, the long-standing association of chronic cough with TB and HIV/AIDs in our study settings was a major driver of stigma and social exclusion experienced by people with CRD and their families. This highlights the negative role of these chronic stigmatizing diseases in framing perceptions and attitudes towards other illnesses in the community. Context-appropriate social safety nets and systematic community health education and sensitization would be required to address the economic and social impacts of CRD identified in this study, as well as the broad causes and management of chronic cough in the communities. In addition, provision of available, accessible, and affordable care for CRD is necessary to break the cycles of poverty, ill-health, and stigma in these sub-Saharan African contexts.

## Supplementary Information

**Additional file 1.** Topic Guide Five: Interview Topic Guide for in-depth interviews with community members (including those affected by CLD).

**Additional file 2**. Topic Guide Seven: Interview Topic Guide for known CRD Patients (EPILAB Sites).

**Additional file 3.** Topic Guide Four: FGD Topic Guide for Community Members.

## Data Availability

The datasets used and/or analysed during the current study are available from the corresponding author on reasonable request.

## Abbreviations

COPD: Chronic obstructive pulmonary disease
COVID: Coronavirus disease
CRD: Chronic respiratory disease
EPILAB: Epidemiological laboratory
FGD: Focus group discussion
HIV/AIDS: Human immunodeficiency virus / Acquired immune deficiency syndrome
IMPALA: International Multidisciplinary Programme to Address Lung Health and TB in Africa
LMIC: Low- and middle income countries
NIMR: National Institute for Medical Research
MOH: Ministry of Health
MOHCDEC: Ministry of Health, Community Development, Gender, Elderly and Children
MSc: Master of science
NTLP: National Tuberculosis and Leprosy Control Programme
PhD: Doctor of Philosophy
SSA: Sub Saharan Africa
TB: Tuberculosis
