# Supplementary material for: “Honestly, this problem has affected me a lot”: a qualitative exploration of the lived experiences of people with chronic respiratory disease in Sudan and Tanzania
Source: BMC Public Health. 2023 Mar 13;23:485. doi: 10.1186/s12889-023-15368-6 (PMC10010645; doi:10.1186/s12889-023-15368-6)
Supplement: Supplementary file 1 — Additional file 1. Topic Guide Five: Interview Topic Guide for in-depth interviews with community members (including those affected by CLD). [file 12889_2023_15368_MOESM1_ESM.docx]

Topic Guide Five: Interview Topic Guide for in-depth interviews with community members (including those affected by CLD)

IDI ID NO: ______________ Facilitator Initials: ___________ Note-taker Initials: __________

Participant group________________ Number of participants___________ Audio file ID _________

Country/Community: _____________________

Where do you work:______________________

Are you married:_________________________

Do you have children:_____________________

Date of IDI _____________________

***Community understanding of CLD***

- What does it mean to have healthy lungs?
- What does it mean if a person does not have healthy lungs?
- In your community is there a word/ term for diseases that affect the lungs over a long time?
- If yes, what is the word?
- Probe what this word means
- I understand that you have experienced lung problems. Can you tell me more about the symptoms you have? How long have you had these symptoms?
- What do you think are the main causes of your lung health problems?

***Impact of CLD on quality of life***

- How has your lung problem influenced your daily activities?
- Probe: what activities can you do/ not do; self-care; community participation; livelihood activities; mobility
- How do you feel about your lung problem?
- Probe: impact on mental wellbeing
- Who are you able to talk to about how you feel?
- Has your lung problem affected your household finances? How?
- Probe: for impact on household spending? Influence on livelihood activities (if not discussed above)? Cost of care seeking? Medicines?
- Can you tell me how you cope with the changes that your lung problem has brought to your life?
- Do you know anyone else in your community living with lung health problems?
- If yes, how do you interact with them? Are you able to talk to them about how you feel?

***Community care-seeking for CLD***

- What did you do when you started to develop these symptoms related to your lung health problem? Probe home remedies, care seeking with traditional healer, care seeking at health facility
- Why did you decide to use home remedy/go to a traditional healer/ seek care at drug shops, pharmacies or a health facility?
- (If attended health facility). Please tell me more about what happened when you went to the health facility. Probe investigations, management, perception of quality/ effectiveness
- (If attended health facility). What was good or bad about the services you got at the health facility? Probe trust in services
- (If didn’t attend health facility). Why did you decide not to attend health facility?
- (If didn’t attend health facility). What would make you more likely to attend the health facility for your lung problems? Why?
- Why would people with lung disease not attend the health facility?
- What do you think would make it easier/ more helpful for them to attend?

***Community priorities for care for CLD***

- What services do you think should be available for someone with lung problems? Probe why?
- Is there anything else that could be done to help you?
- Probe for: physically, mentally, socially, medically
- Why?
- If someone is trained in your community to know the signs and symptoms of CLD and has the capacity to refer you to the nearest health facility, would that help? Why? Why not?
- What would you think if the health centre/district hospital was able to do a test to find out what was causing your lung problems? Why?
- What would you think if the health centre/district hospital was able to write a prescription and provide medicine that would help you feel better if you kept taking it regularly? Why?

**Community experiences with community TB services (Tanzania only)**

- (Tanzania only). Have you told your CHW about your lung problem? Why? Why not?
- (Tanzania only). Can the CHW help you to get health services for your lung problem? Why? Why not?
- (Tanzania only). How could the CHW help you more with your lung problem?

26. What would you think about CLD services being integrated with TB services? Would you be willing to receive care through the TB programme? Why/Why not?
