# Supplementary material for: “Honestly, this problem has affected me a lot”: a qualitative exploration of the lived experiences of people with chronic respiratory disease in Sudan and Tanzania
Source: BMC Public Health. 2023 Mar 13;23:485. doi: 10.1186/s12889-023-15368-6 (PMC10010645; doi:10.1186/s12889-023-15368-6)
Supplement: Supplementary file 2 — Additional file 2. Topic Guide Seven: Interview Topic Guide for known CRD Patients (EPILAB Sites). [file 12889_2023_15368_MOESM2_ESM.docx]

Topic Guide Seven: Interview Topic Guide for known CRD Patients (EPILAB Sites)

IDI ID NO: ______________ Facilitator Initials: ___________ Note-taker Initials: __________

Participant group________________ Number of participants___________ Audio file ID _________

Country/Community: _____________________

Where do you work:______________________

Are you married:_________________________

Do you have children:_____________________

Date of IDI _____________________

***Community understanding of CLD***

- What does it mean to have healthy lungs?
- What does it mean if a person doesn’t have health lungs?
- What did the doctor / health worker tell you about your illness / condition when you were diagnosed?

***Impact of CLD on quality of life***

- I understand that you have experienced asthma. Can you tell me more about the symptoms you have? How long have you had this condition?
- How has asthma influenced your daily activities?
- Probe: what activities can you do/ not do; self-care; community participation; livelihood activities; mobility
- How do you feel about living with asthma?
- Probe impact on mental wellbeing
- Who are you able to talk to about how you feel?
- Has your asthma affected your household finances? How?
- Probe: for impact on household spending? Influence on livelihood activities (if not discussed above)? Cost of care seeking? Medicines?
- How much money do you spend per month on accessing medicines/treatment for asthma?
- Can you tell me how you cope with the changes that asthma has brought to your life?
- Do you know anyone else in your community with the same problem as you?
- If yes, how do you interact with them? E.g. talking/sharing of medicines etc.
- Are you able to talk to them about how you feel?

***Community care-seeking for CLD***

- What did you do when you started to develop these symptoms?
- Probe home remedies, care seeking with traditional healer, care seeking at health facility
- Why did you decide to seek care at a health facility?
- Please tell me more about what happened when you went to the health facility.
- Probe investigations, management, perception of quality/ effectiveness
- What was good about the services you got at the health facility?
- What was not good about the services you got at the health facility?
- What steps do you take now when you start to experience symptoms of asthma/asthma attack?
- How often does this occur?

***Community priorities for care for CLD***

- How can the asthma management services at the health facility be made better?
- What do you think are the main priorities for health services for people with asthma?
- What would make it easier to cope with your asthma? Why?
- Probe for: physically, mentally, socially, medically
- Is there anyone in your community who has asthma who does not attend the health facility?
- Why do you think he/she does not attend?
- What do you think would make it easier/ more helpful for that person to attend?
- What could be done within your community to make asthma management easier?
- Prompt for: community support groups; links to community health workers/other health providers etc.
