# Supplementary material for: “Honestly, this problem has affected me a lot”: a qualitative exploration of the lived experiences of people with chronic respiratory disease in Sudan and Tanzania
Source: BMC Public Health. 2023 Mar 13;23:485. doi: 10.1186/s12889-023-15368-6 (PMC10010645; doi:10.1186/s12889-023-15368-6)
Supplement: Supplementary file 3 — Additional file 3. Topic Guide Four: FGD Topic Guide for Community Members. [file 12889_2023_15368_MOESM3_ESM.docx]

Topic Guide Four: FGD Topic Guide for Community Members

FGD ID NO: ______________ Facilitator Initials: ___________ Note-taker Initials: __________

Participant group________________ Number of participants___________ Audio file ID _________

Country/Community: _____________________ Date of FGD _____________________

***Community understanding of CLD***

- What are the main diseases in this area? (Probe for reasons)

***In our discussion today, we are going to focus on lung health and chronic lung diseases such as asthma, COPD, occupational lung disease and TB.***

- What does it mean to have healthy lungs?
- What does it mean if a person does not have healthy lungs?
- In your community is there a word/ term for diseases that affect the lungs over a long time?
- If yes, what is the word/term?
- Probe what this word/term mean
  - 1. What are the general symptoms of lung health problems?
    2. What activities or practices in the community cause lung health problems?
- How might lung problems affect someone’s life? Probe what activities someone might experience challenges with or might not be able to do
  probe (self-care; community participation; livelihood activities; mobility)
- What are the community attitudes and perceptions towards people with chronic lung disease problems?

***Community care-seeking for CLD***

- If someone in your community has lung problems, what might he/she do to manage them?
- Probe home remedies, care seeking with traditional healer (Spiritual healer, traditional medicine, others), care seeking at health facility, go directly to the pharmacy
- Why might someone with lung problems decide to use home remedy/go to a traditional healer/ seek care at a health facility?
- Please tell me more about the services provided at the health facility
- Probe what do you like about them?
- What could be improved about the services?
- Probe trust in existing services. (availability, quality, interaction with health provider, confidentiality, treatment outcome)
- What services do you think should be available for someone with lung problems? probe for differences between health centre and hospital, public and private
- Probe why?

***Community priorities for care for CLD***

- What would make it easier for someone with lung problems to cope? Why?
- Is there anyone in your community who has lung problems who does not attend the health facility?
- Why do you think he/she does not attend?
- What do you think would make it easier/ more helpful for that person to attend? (probe for diagnostic facilities, medicine, enough staff, good client provider relationship, cost)
- If the CHW or a health worker (trained volunteer) was able to refer someone with lung problems to the health centre/district hospital, would that make a difference? Why? Why not?
- What would you think if the health centre/district hospital was able to do a test to find out what was causing lung problems? Why?
- What would you think if the health centre/district hospital was able to write a prescription for medicine that would help someone with lung problems feel better if he/she kept taking it regularly? Why?
